# Alcohol-medication interactions among older people: a narrative review on age-specific evidence

**DOI:** 10.1093/alcalc/agag018

**Published:** 2026-04-05

**Authors:** Francesco Salis, Maristella Belfiori, Roberta Agabio, Antonella Mandas

**Affiliations:** Department of Medical Sciences and Public Health, University of Cagliari, S.P. Monserrato-Sestu Km 0,700, Monserrato, Cagliari, 09042, Italy; Department of Biomedical Sciences, Section of Neuroscience and Clinical Pharmacology, University of Cagliari, S.P. Monserrato-Sestu Km 0,700, Monserrato, Cagliari, 09042, Italy; Department of Medical Sciences and Public Health, University of Cagliari, S.P. Monserrato-Sestu Km 0,700, Monserrato, Cagliari, 09042, Italy; Department of Biomedical Sciences, Section of Neuroscience and Clinical Pharmacology, University of Cagliari, S.P. Monserrato-Sestu Km 0,700, Monserrato, Cagliari, 09042, Italy; Department of Medical Sciences and Public Health, University of Cagliari, S.P. Monserrato-Sestu Km 0,700, Monserrato, Cagliari, 09042, Italy

**Keywords:** AMI, alcohol use, geriatrics, pharmacology

## Abstract

**Aims:**

Alcohol use is relatively common in older adults, representing a challenge for geriatricians. Older people are also prone to chronically taking multiple medications. Concerns are raised about potential alcohol-medication interactions (AMIs). This review aims to summarize current evidence on AMIs in older adults.

**Methods:**

We conducted a literature search using MEDLINE up to 18 June 2025. Of 481 identified studies, 37 were considered pertinent and discussed in this review.

**Results:**

Epidemiological data reveal that slightly over 25% (from ~20% to 80%) of older adults concurrently take alcohol and potentially interact with medications. Negative effects related to potential AMIs range from excessive sedation when old people take benzodiazepines, opioids, or antipsychotics, to hypoglycemia when they take sulfonylureas. Pharmacokinetic modifications can depend on physiological age-related changes in the human body. This evidence mostly derives from studies not focused on older adults.

**Conclusions:**

Despite their clinical importance, few studies focus on AMIs in older adults. The limited awareness among both healthcare providers and patients represents a significant public health issue. Routine alcohol use screening, universally shared lists of potential AMIs, and larger longitudinal studies are necessary to further explore the theme and mitigate risks.

## Teaser

**Short summary** On average, over 25% (range: 20%–80%) of older adults take alcohol together with interacting medications, especially those acting on the central nervous system. Alcohol-medication interactions may increase the risk of negative medication effects—such as excessive sedation and hypoglycemia—and falls. Physicians should advise their older patients on these risks.

## Introduction

Alcohol is a commonly consumed psychoactive substance, and its use represents a significant public health issue across all age groups, including older adults (Sudhinaraset et al. 2016, MacKillop et al. 2022, White et al. 2023). It is estimated that ~43% of US residents aged 65 years and older are current alcohol users and that the annual increase in the rate of people using alcohol is faster among older adults than in younger individuals (National Survey on Drug Use and Health, 2020; White et al. 2023, Haight et al. 2024).

Among the general population, the amount of alcohol consumed is directly linked to the risk of developing negative consequences, with the lowest risk corresponding to no use (GBD 2016 Alcohol Collaborators 2018). Considering the widespread use of alcohol, recommendations for “at low-risk” consumption have been adopted to help the population adopt safer lifestyles. In these recommendations, alcohol use is measured in standard drinks also known as “alcoholic units” (AUs), a common measure with large variability in the amount of pure alcohol among countries (NIAAA 2005, MacKillop et al. 2022). In Europe, an AU usually corresponds to 10–14 g. Using this unit of measure, for healthy men, recommendations for at low-risk alcohol use should not exceed two AUs a day for people who drink daily (and no more than four AUs on a single occasion for people who drink occasionally), while for healthy non-pregnant, non-breastfeeding, adult women, this threshold is no more than one AU a day for people who drink daily (and no more than three AUs on a single occasion for people who drink occasionally; NIAAA 2005, MacKillop et al. 2022). In some situations, any amount of alcohol use is risky like, for instance, during pregnancy (Minozzi et al. 2025) or when people are taking medications that can potentially interact with alcohol (Traccis et al. 2022).

There is currently no universally accepted consensus regarding the appropriate methodologies for estimating alcohol consumption or thresholds for low-alcohol use among older adults (Tevik et al. 2021). This lack of standardization is particularly concerning, as research indicates that even low levels of alcohol intake may be linked to adverse health outcomes (Ortolá et al. 2024).

As an example, the different recommendations for men and women are since following the consumption of similar amounts of alcohol, women reach higher blood alcohol concentrations than men (due to differences in body composition, body weight, and activity of the gastric enzyme alcohol dehydrogenase, ADH) (Agabio et al. 2017). However, these differences gradually reduce with age and disappear among older people (NIAAA 2005, MacKillop et al. 2022). Accordingly, thresholds for low-risk alcohol use may not require gender or sex differences among older people (Tevik et al. 2021).

Exceeding established thresholds of alcohol intake significantly increases the risk of alcohol-related negative consequences (NIAAA 2005, MacKillop et al. 2022), particularly in older adults who may experience amplified effects due to coexisting illnesses and pharmacotherapy (NIAAA 2005, Holton et al. 2017a). When people lose the ability to control alcohol consumption, they may develop alcohol use disorder (AUD), a severe and frequent mental disorder characterized by impaired regulation of consumption despite harmful consequences (APA 2022). AUD presented a multifactorial etiology, including both genetic, psychological, and environmental influences, with the heritability being estimated up to 60% (MacKillop et al. 2022). AUD can benefit from a treatment approach that combines medications and psychological interventions (Lehner et al. 2024, Minozzi et al. 2025).

In the USA, ~15% of older adults meet AUD criteria during their life (Grant et al. 2015). Moreover, prevalence and mortality of AUD and alcohol-associated liver disease (ALD) have been rising among older US adults during the last 20 years (Danpanichkul et al. 2025). Over the past two decades, the annual percent change (APC) in AUD prevalence was estimated at 0.54%, while the APC in AUD-related mortality reached 1.34%. In 2021, 56 990 cases of ALD and 5930 ALD-related deaths were reported. In Europe, a quarter of people aged 50 years or more are “hazardous users of alcohol” (i.e. consuming more than six AUs per single occasion and/or more than 12.5 AUs per week; Tschorn et al. 2023).

Due to its sedative effects, acute alcohol use can exacerbate age-related changes in balance and cognition, increasing the risk of falls (Xu et al. 2022), and fall-related injuries (Sheahan et al. 1995, Xu et al. 2022). Consequently, such incidents frequently lead older adults to emergency department visits and subsequent hospitalizations, thereby adding to the overall healthcare burden (Dharia and Slattum 2011, White et al. 2023). Also, chronic use worsens sleep disorders and increases the risk of various health issues, such as liver diseases, cancers, systemic hypertension, cardiovascular diseases, and cognitive impairment (Moore et al. 2007, White et al. 2023, Zahr 2024, Zarezadeh et al. 2024, Anton et al. 2025). Alcohol-related liver disease and liver cancer have notably increased among older adults in recent decades (Danpanichkul et al. 2025), while alcohol misuse throughout the lifespan has been proposed as a modifiable risk factor for Alzheimer’s disease and related dementias (Anton et al. 2025).

While its relationship with geriatric syndromes such as sarcopenia and frailty remains a subject of debate (Soltani et al. 2024, Mao et al. 2025), alcohol use in later life may be linked to unhealthy dietary patterns, thus increasing the risk of malnutrition (Cruz-Jentoft and Volkert 2025).

Both acute and chronic alcohol consumption can lead to alcohol-medication interactions (AMIs), when alcohol is consumed by people taking medications (Heuberger 2009, Johnson and Seneviratne 2014, Traccis et al. 2022). Alcohol does not just interact with medications, but it can also be affected by them, creating a complex area of concern. AMIs include alterations in the pharmacokinetics (absorption, distribution, metabolism, and elimination) and pharmacodynamics of both medications and alcohol (Moore et al. 2007, Traccis et al. 2022). In other words, they can either enhance or diminish the therapeutic effects of medications or even exacerbate their side effects as well as increase alcohol blood levels, thereby intensifying alcohol-related negative consequences. “Polypharmacy,” which affects more than one in three older adults (Delara et al. 2022), refers to the chronic use of five or more different medications simultaneously and represents a major contributor to medication–medication interactions and AMIs (Adams 1995a, Gorsen et al. 2021). Despite growing awareness on the topic, the extent of their impact remains a subject of debate. To the best of our knowledge, although some articles are available on AMIs in the general population (Traccis et al. 2022), the first systematic review that addresses the prevalence and the impact of adverse outcomes of AMIs specifically in older adults was published in 2017, denouncing a lack of consensus among researchers and clinicians on alcohol-interacting medications, and lacking prospective studies (Holton et al. 2017a). Since then, some further studies have been published on AMIs specifically focusing on older adults (Holton et al. 2019a, Holton et al. 2019b, Gorsen et al. 2021, Schröder et al. 2024).

While much research has focused on medication–medication interactions, AMIs are less explored in geriatric medicine. The aim of this narrative review is to summarize the available evidence of the epidemiological and clinical impact of AMIs on older adults.

## Materials and methods

Data were obtained by searching for published medical literature in MEDLINE via PubMed from inception up to 18 June 2025. The search strings used were the following: (“ethanol”[MeSH Terms] OR “ethanol”[All Fields] OR “ethanols”[All Fields] OR “ethanol s”[All Fields] OR “ethanolic”[All Fields]) AND (“aged”[MeSH Terms] OR “aged”[All Fields]) AND (“drug interactions”[MeSH Terms] OR (“drug”[All Fields] AND “interactions”[All Fields]) OR “drug interactions”[All Fields]), and (“ethanol”[MeSH Terms] OR “ethanol”[All Fields] OR “ethanols”[All Fields] OR “ethanol s”[All Fields] OR “ethanolic”[All Fields]) AND (“aged, 80 and over”[MeSH Terms] OR “80 and over aged”[All Fields] OR “aged 80 and over”[All Fields]) AND (“drug interactions”[MeSH Terms] OR (“drug”[All Fields] AND “interactions”[All Fields]) OR “drug interactions”[All Fields]), with the filters “Humans” and “English.” In addition to the references retrieved through this search strategy, we have also included articles from the references of the individual papers cited. Studies were classified as “unrelated,” and excluded from further analysis, if they did not address AMIs in older adults. Finally, we classified and described the results of the included studies according to their main topics into the following sections: epidemiology, pharmacokinetics, and clinical aspects. These results are based on evidence from peer-reviewed studies and empirical data concerning the impact of potential AMIs on older adults.

## Results

**Figure 1:**
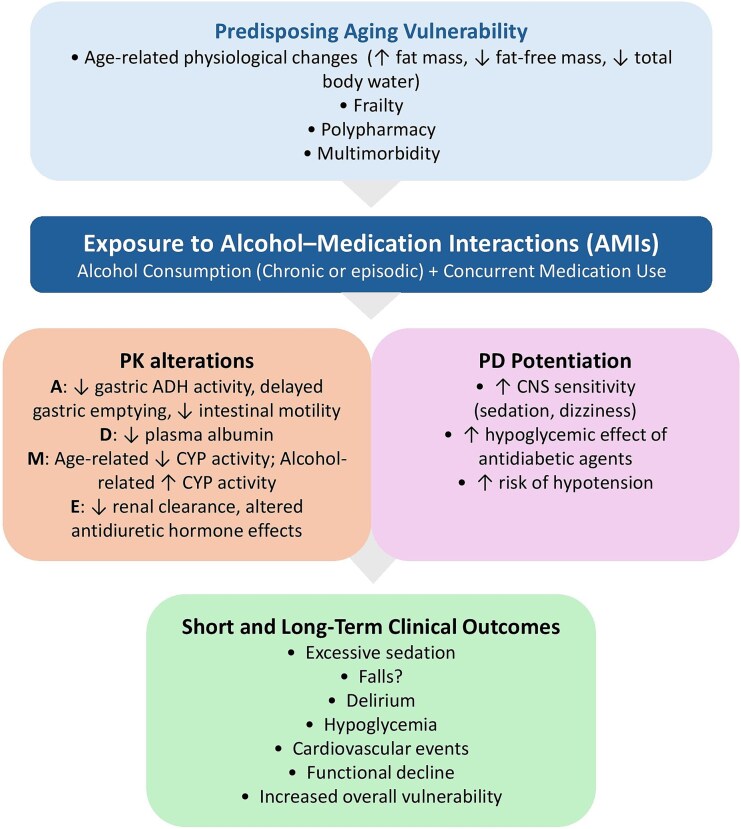
Conceptual life-course framework of AMIs in older adults. Conceptual life-course framework illustrating how age-related factors increase vulnerability to AMIs, affecting pharmacokinetics and pharmacodynamics and leading to adverse clinical outcomes. Abbreviations, A, absorption; ADH, alcohol dehydrogenase; CNS, central nervous system; CYP, cytochrome P450; D, distribution; E, elimination; M, metabolism; PK, pharmacokinetics; PD, pharmacodynamics.

Our search produced 462 results, to which we added 19 papers from the reference lists of the identified results and from the lists of recent studies that cite the papers already included, for a total of 481 studies. Altogether, 444 studies were excluded as unrelated, and 43 studies were included and discussed in this work. The main findings of the included studies are summarized in Supplementary Table S1 (in which studies are in alphabetical order according to the names of the first authors), and a conceptual life-course framework of AMIs in older adults is presented in Fig. 1.

### Epidemiological findings

Tables 1 and 2 show a summary of our epidemiological findings.

**Table 1:** Summary of studies that reported AMIs prevalence.

Study	Country	Participants	Age (years)	Subtype of medications	Assessment of alcohol use	AMIs prevalence (%)
Adams (1995b)	United States	311	Mean: 83	–	Questionnaire	38.0
Breslow et al. (2015)	United States	7183	65+	–	Self-reporting	77.8
Cousins et al. (2014)	Ireland	3815	Mean: 70	–	Self-reporting	60.0
Del Río et al. (2002)	Spain	1025	66+	Benzodiazepines	Self-reporting	Subgroup of 352 people aged 75+ years: 15.9
Du et al. (2008)	Germany	1605	60 to 79	Psychotropic medications	Self-reporting	7.6
Forster et al. (1993)	United States	667	Mean: 74	–	Self-reporting	25.0
Holton et al. (2019a)	Ireland	1459	Mean: 72	List by Holton et al. (2017b)	Self-reporting	Severe AMIs: 18
Ilomäki et al. (2013)	Australia	1705	70+	Psychotropic medications	Self-reporting	Antidepressants: 27.0; Anxiolytics: 43.0
Immonen et al. (2013)	Finland	1395	Mean: 79	–	Self-reporting	Moderate alcohol use: 35.0
Lagnaoui et al. (2001)	France	3767	Mean: 75	Benzodiazepines	Questionnaire on food habits	Moderate alcohol use: 31.3; heavy alcohol use: 19.3
Pringle et al. (2005)	United States	83 321	Mean: 79	–	Self-reporting	19.0
Qato et al. (2015)	United States	1960	Mean: 79	–	Self-reporting	20.0
Schröder et al. (2024)	Germany	114	65+	List by Holton et al. (2017b)	NA (AUD was an inclusion criteria)	Severe AMIs: 80.7

AMIs, alcohol-medication interactions; AUD, alcohol use disorder; NA, not available; –, not applicable.

**Table 2:** Main findings of the included studies.

Domain	Emerging findings
Epidemiology	High and persistent prevalence of concurrent alcohol and prescription drug use in adults ≥60–65 years; Substantial exposure to potentially serious AMIs (e.g., POSAMINO criteria); Sociodemographic predictors of co-use (male sex, younger-old age, higher education, multimorbidity, polypharmacy); Increasing alcohol–medication-related hospitalizations ≥50 years; Absence of a clearly safe alcohol threshold in the presence of pharmacotherapy.
Pharmacology	Age-related pharmacokinetic changes leading to increased drug half-life and toxicity; Alcohol-induced CYP modulation (CYP2E1 induction, altered CYP2D6, and CYP2C19 activity); Mixed PK-PD mechanisms underlying CNS depression, hypoglycemia, hypotension, and hepatotoxicity; Limited age-specific interaction data.
Clinical findings	Frequent CNS-related AMIs associated with sedation, cognitive impairment, falls, and injurious outcomes; Synergistic GI toxicity with NSAIDs/aspirin; Increased risk of adverse drug reactions (metabolic/endocrine, cardiovascular); High prevalence of contraindicated medication use among heavy drinkers; Recommendation for routine alcohol screening (e.g., CAGE, MAST-G) in older adults receiving high-risk medications.

AMIs, alcohol–medication interactions; CAGE, cut-down, annoyed, guilty, eye-opener questionnaire; CNS, central nervous system; CYP, cytochrome P450; GI, gastrointestinal; MAST-G, Michigan Alcoholism Screening Test–Geriatric Version; NSAIDs, nonsteroidal anti-inflammatory drugs; PK, pharmacokinetics; PD, pharmacodynamics; POSAMINO, potentially serious alcohol–medication interactions in older adults

According to a systematic review, between 1:5 and 1:3 older people are at risk for potential AMIs (Holton et al. 2017a). Some years ago, a research group developed a list of serious AMIs through expert consensus (Holton et al. 2017b) and estimated that 18% of adults aged 65 years and older are affected by these serious AMIs (Holton et al. 2019a; see Table 1). However, prevalence of AMIs largely varies across studies (Holton et al. 2017a, Schröder et al. 2024). We found some cross-sectional studies that investigated the epidemiology of AMIs related to different medication classes. One of these studies showed that around 20% of nearly 2000 US subjects simultaneously took alcohol and potentially interacting medications (Qato et al. 2015). This study was devoted to older subjects but also included people aged 57 years or older (Qato et al. 2015). A similar rate (19%) was reported by an observational study that analyzed more than 83 000 US subjects (mean age: 79 years) from the Pennsylvania Pharmaceutical Assistance Contract for the Elderly program (Pringle et al. 2005). Other studies reported higher rates, starting from 25% according to a cross-sectional study (667 older subjects; mean age:74 years; Forster et al. 1993), through the 38% according to a study conducted in the USA (311 participants; mean age: 83 years; Adams 1995b), to the 60% according to an Irish cross-sectional study (more than 3800 subjects; mean age: 70 years; Cousins et al. 2014). This rate reached 77.8% according to a cross-sectional study conducted in the USA (more than 7100 subjects aged 65 years or older; Breslow et al. 2015). Some other studies with similar designs did not clearly report the rate of people taking alcohol and potentially interacting medications (Del Río et al. 1996, Aira et al. 2005, Veldhuizen et al. 2009, Wong et al. 2016, Gorsen et al. 2021) or described data including significantly younger people (Ilomäki et al. 2008).

We also found a series of European observational studies that investigated interactions regarding certain pharmacological classes, particularly those acting on the central nervous system (CNS). A Spanish study (more than 1000 subjects; aged 66 years and older) reported that almost 16% of the patients aged 75 years or older took benzodiazepines and alcohol (Del Río et al. 2002). A French study with a similar design (more than 3700 subjects; mean age: 75 years) found a slightly higher percentage when considering heavy alcohol consumption (19.3%), and significantly higher (31.3%) when considering moderate alcohol consumption (Lagnaoui et al. 2001). According to a German survey, 7.6% of the interviewed (1605 people; aged 60 years or older) had a last-week prevalence of combined psychotropic medications and alcohol use (Du et al. 2008). Finally, higher rates were found by an Australian study (1774 people; aged 70 years and older), where 27% of people taking antidepressants, and 43% of people taking anxiolytic medications, also took alcohol (Ilomäki et al. 2013).

### Pharmacokinetics

Age- and alcohol-related alterations profoundly reshape drug pharmacokinetics, ultimately redefining therapeutic efficacy and safety (Bebia et al. 2004; see Table 2 and Supplementary Table S1).

It is well established that alcohol is absorbed primarily through the upper gastrointestinal tract, where it is partially metabolized by the gastric enzyme ADH, and it is mainly metabolized in the liver (Meier and Seitz 2008). The activity of ADH decreases with age, and this reduction of its activity potentially increases the amount of absorbable alcohol (Anderson et al. 2012).

Delayed gastric emptying, together with reduced intestinal motility, is a common finding in the aged gastrointestinal system (Adams 1995a, Fraser 1997, Moore et al. 2007). These changes can slow the absorption of orally administered compounds (Moore et al. 2007). In addition, older people’s body mass usually differs from the younger adults’, resulting in increased fat mass and decreased fat-free mass (i.e. muscle) and total body water, even considering sex-related differences (Adams 1995a, Rigler 2000, Moore et al. 2007).

Also, compared to younger people, as alcohol is hydrophilic (due to its –OH group), it tends to reach higher concentrations at the same dose among older people (Weathermon and Crabb 1999). As a result, lipophilic compounds tend to have a higher volume of distribution in older adults, whereas hydrophilic ones have a lower volume of distribution. As for distribution, compared to younger people, older people usually have decreased albumin levels due to lower hepatic synthetic capacities (Rigler 2000). It may result in lower binding of acid compounds and a higher active compound availability (Moore et al. 2007).

The reduced synthetic capacities mentioned are the result of reduced liver mass, hepatic blood flow, and enzyme activity, which can also reduce medications’ metabolism and clearance (Moore et al. 2007, Meier and Seitz 2008), therefore resulting in higher blood concentrations (Adams 1995a). Similarly, compared to younger people, older people show a lower glomerular filtration rate, reflection of lower renal flow, for compounds excreted by the kidneys (Moore et al. 2007). Therefore, aging itself is characterized by changes in body composition and organ physiology, including delayed gastric emptying, reduced hepatic blood flow, and diminished synthetic capacity, which are further exacerbated by alcohol use.

Our review highlighted that these combined age- and alcohol-related pharmacokinetic alterations constitute a central mechanistic substrate of AMIs in older adults, particularly in the presence of polypharmacy and medications with narrow therapeutic indices.

In addition, alcohol consumption increases intestinal transit time (Rigler 2000), thereby modifying the absorption of both alcohol and medications. As an example, even if the clinical consequences do not appear to be dramatic, the delayed gastric emptying enhances the absorption rate of benzodiazepines (Adams 1995a). On the other hand, H2-inhibitors inhibit the activity of gastric ADH, resulting in higher absorption and higher alcohol blood levels (Adams 1995a, Moore et al. 2007).

At the hepatic level, chronic alcohol consumption may induce cytochrome p450 enzymes, especially CYP2E1, despite the age-related decline in liver function (Adams 1995a, Rigler 2000, Lu and Cederbaum 2018). CYP2E1 induction led to enhanced production of reactive oxygen species, promoting DNA damage, and altered metabolism of ethanol and other xenobiotics (Bebia et al. 2004, García-Suástegui et al. 2017). Chronic alcohol exposure increases ADH III activity in the brain, leading to modest local acetaldehyde production (Silva-Adaya et al. 2021). Epigenetic changes, such as reduced methylation of the CYP2E1 gene in older adults and patients with Parkinson’s disease, may further modulate its metabolic activity (Silva-Adaya et al. 2021). For instance, acetaminophen—that is widely used among older adults—is metabolized by CYP2E1 (Weathermon and Crabb 1999). The induction of CYP2E1 due to alcohol use can result in higher concentration of the metabolite N-acetyl-p-benzoquinone imine of acetaminophen, a metabolite that is harmful to the liver (Weathermon and Crabb 1999).

Therefore, alcohol influences the metabolism of frequently used medications, i.e. benzodiazepines, warfarin, and propranolol (Moore et al. 2007, Dharia and Slattum 2011). Paradoxically, while chronic alcohol use induces hepatic enzymes, short-term consumption of significant amounts of alcohol inhibits hepatic metabolism, probably due to competition for cytochrome p450 enzymes (Fraser 1997), thus increasing blood concentrations of medications such as benzodiazepines and warfarin (Moore et al. 2007). As an example, a recent systematic review found that among participants taking medications like diazepam, cannabis-based pharmaceutical products, and opioids, those who consumed alcohol had higher blood concentrations of these medications compared to those who did not consume alcohol (Traccis et al. 2022).

Another key enzyme affected by both aging and alcohol is CYP2D6, which metabolizes dopamine, serotonin, and numerous centrally active drugs, including antidepressants, antipsychotics, and analgesics (Miksys et al. 2002, Mann et al. 2012). CYP2D6 expression increases in adulthood, potentially reducing local drug concentrations and attenuating therapeutic response (Mann et al. 2012). Chronic alcohol consumption further elevates brain CYP2D6 expression (Kirchheiner et al. 2011), heightening susceptibility to neurotoxicity and contributing to the increased incidence of AMI in people with AUD (Miksys et al. 2002).

As for excretion, prolonged alcohol exposure inhibits the release of antidiuretic hormone due to actions on hypothalamic neurons (Silva et al. 2002). This effect can theoretically increase the elimination of medications with renal excretion, even if with doubted clinical relevance (Adams 1995a).

These pharmacokinetic modifications may be particularly concerning in people using medications with narrow therapeutic indexes (Rigler 2000), or those that produce toxic metabolites, such as the abovementioned acetaminophen.

Among different active principles belonging to the same pharmacological class, there can be some differences in alcohol-induced pharmacokinetic modifications. As an example, alcohol can reduce diazepam clearance (which can also be reduced in physiological aging), while it does not significantly alter lorazepam pharmacokinetics (Meier and Seitz 2008, Traccis et al. 2022).

However, most of the existing knowledge on alterations in alcohol/medications pharmacokinetics in relation to AMIs derives from deductions based on clinical or preclinical assumptions, rather than direct evidence on older subjects (Adams 1995a, Anderson et al. 2012, Traccis et al. 2022).

### Clinical issues

Age- and alcohol-induced alterations in CYP450 activity and related metabolic pathways increase the formation of neurotoxic intermediates, modify the pharmacokinetics and clinical efficacy of psychoactive medications, and reduce the brain’s capacity to detoxify xenobiotics and endogenous metabolites (Ravindranath and Boyd 1995, Silva-Adaya et al. 2021).

We found a limited number of clinical studies investigating potential negative effects deriving from AMIs, mainly related to medications acting on the CNS (Holton et al. 2017a). In this section, we briefly describe the results of these studies. These results are summarized in Tables 2 and 3.

**Table 3:** Summary of common AMIs and potential negative effects in older people.

ATC group	Medication	Possible interactions with alcohol	Studies in older people	Potential negative effects in older people
A (alimentary tract and metabolism)	Sulfonylureas	Variations in insulin-sensitivity	Yes	Chronic alcohol use: hypoglycemia
C (cardiovascular)	Beta-blockers	NA	Yes	Higher heart rate and systolic blood pressure
	Calcium channel blockers	NA	Yes	Higher systolic blood pressure
	Statins	Shorter half-life	Yes	Higher blood lipid levels
G (genito-urinary)	Phosphodiesterase type 5 inhibitors	Higher blood concentrations	No	Cardiovascular side effects
J (anti-infective)	Antibiotics	Beta-lactams: longer half-life; tetracyclines: variations in blood concentrations	No	Disulfiram-like reactions
M (musculo-skeletal)	NSAIDs	NA	Yes	Gastrointestinal bleeding
N (nervous)	Benzodiazepines	Higher blood concentrations	Yes	Excessive sedation
	Opioids	Shorter half-life	Yes	Rapid and excessive sedation
	Antidepressants	SSRIs and trazodone: longer half-life; bupropion: lower blood concentrations	Yes	Increased rate of antidepressants’ side effects (e.g. insomnia, anxiety, nausea)
	Antipsychotics	Atypical antipsychotics: longer half-life	Yes	Excessive CNS depression

ATC, anatomical therapeutic chemical classification; CNS, central nervous system; NA, not available; NSAIDs, non-steroidal anti-inflammatory drugs; SSRIs, selective serotonin reuptake inhibitors

A US retrospective study analyzed hospital records to estimate hospitalization rate due to AMIs in individuals aged 50 years and older (Zanjani et al. 2016). The authors identified over 2100 hospitalizations over a 12-year period, finding that the hospitalization rate doubled over the years, from <10 to nearly 20 per 100 000 people in the corresponding population (Zanjani et al. 2016). CNS-acting medications (N, Nervous according to the Anatomical Therapeutic Classification, ATC; Table 3) were the most involved, especially benzodiazepines, usually taken together with opioids (Zanjani et al. 2016). An Italian cross-sectional study (almost 23 000 patients admitted to acute care hospitals; mean age: 70 years) demonstrated that moderate alcohol consumption (defined as the intake of ≤40 g of alcohol/day) significantly increased the likelihood of adverse drug reactions (ADRs; odds ratio 1.24), especially among women. It also raised the frequency of metabolic complications (odds ratio 1.67; Onder et al. 2002).

A systematic review focusing on AMIs in older adults, which included 20 studies, showed that psychotropic medications were the most studied alcohol-interacting medications. Almost 8% of people taking psychotropic medications also consumed alcohol (Holton et al. 2017a). This aspect appears to be of great interest, since the combination of alcohol and medications is implicated in significant geriatric concerns, particularly falls, although debated. An older study (over 1000 community-dwelling people aged 55 years and older) proposed a logistic model according to which psychoactive medication use was a predictor of falls, while alcohol use was not (Sheahan et al. 1995). However, this study did not investigate the combined effect of medications and alcohol use. A more recent study (over 2400 community-dwelling people; mean age: 77 years) found similar results (Wong et al. 2016). A cross-sectional Finnish study (almost 1400 subjects; mean age: 79) observed that falls and injuries were more common in people classified as “at risk users” (consuming >7 AUs/week, or ≥5 AUs per drinking day, or ≥3 AUs several times per week) than in people with lower alcohol consumption (Immonen et al. 2013).

Also, we identified a recent longitudinal study focused on potentially serious AMIs in older adults (Holton et al. 2019a). The authors analyzed an Irish population-based survey (almost 1500 community-dwelling participants; mean age: 72 years), over 4 years. They found that the number of potential serious AMIs did not vary over the follow-up period, while a higher number of chronic conditions and medications was associated with increased risk of such interactions, as defined by the potentially serious alcohol–medication interactions in older adults criteria—an explicit list of 38 serious alcohol-medication combinations for older adults (Holton et al. 2019a). Unfortunately, this study only focused on potential results of AMIs, rather than actual incidence of adverse events. Additionally, a subsequent study examined the same cohort specifically focusing on falls (Holton et al. 2019b). The findings indicate that AMIs involving medications acting on CNS were significantly associated with an increased risk of both 4-year self-reported falls (adjusted relative risk 1.50) and injurious falls (adjusted relative risk 1.62; Holton et al. 2019b).

Although CNS-active drugs are the most consistently studied, other ATC categories are implicated (Table 3). In these categories, reported risks include hypoglycemia, gastrointestinal bleeding, cardiovascular instability, and disulfiram-like reactions. No age-specific interaction studies were identified for genito-urinary (G) and anti-infective (J) agents. For cardiovascular drugs (beta-blockers and calcium channel blockers) and NSAIDs, evidence in older adults exists, yet specific data on alcohol-related interactions remain scarce.

## Discussion

This review was aimed at summarizing the impact of potential AMIs on older adults (see Tables 1–3 and Fig. 1).

Across epidemiological studies (Tables 1 and 2), estimates of older adults concurrently using alcohol and potentially interacting medications varied widely, from ~20% to nearly 80%, with an average of 25.1%. This variability highlights the challenge of accurately assessing the scope of the issue, which is compounded by differences in study populations, methodologies, and definitions of alcohol consumption, still representing a significant public health issue (Breslow et al. 2015, Qato et al. 2015). Notably, many studies also include individuals younger than 65 years, which may not fully capture the specific vulnerabilities of the oldest old.

We described the pharmacokinetics alterations due to AMIs (Tables 2 and 3; Fig. 1). However, findings are mostly derived from deductions from studies conducted in populations other than older adults. Hence, the clinical evidence remains debatable (Adams 1995a, Anderson et al. 2012). Anyway, it can be affirmed that the competition between alcohol and various medications for cytochrome p450 enzymes, particularly CYP2E1, results in complex metabolic interactions that can either enhance or diminish the efficacy of drugs (Rigler 2000, Lu and Cederbaum 2018, Traccis et al. 2022). Therefore, individuals’ drinking patterns should always be evaluated considering potential AMIs, especially in older people with polypharmacy.

Clinically, the most frequently studied AMIs involve CNS-active medications, particularly sedatives such as benzodiazepines, opioids, and antipsychotics (Holton et al. 2017a). Concurrent use raises concerns about excessive sedation (Tables 2 and 3), resulting in an increased risk of falls. Nonetheless, it appears that more robust longitudinal studies are needed to clarify the role of alcohol in fall-related adverse events among older adults, as existing findings are conflicting (Sheahan et al. 1995, Immonen et al. 2013, Wong et al. 2016).

Beyond CNS-active agents, other negative effects induced by AMIs regard medications used for the treatment of the most common conditions in older people worldwide, such as diabetes, systemic hypertension, and hypercholesterolemia (Diaz et al. 2021). Alcohol can enhance the hypoglycemic effects of sulfonylureas, diminish the efficacy of antihypertensives such as beta-blockers, and reduce the therapeutic impact of statins with higher blood lipid levels (Holton et al. 2017a, Diaz et al. 2021). These interactions are particularly concerning given the high prevalence of cardiometabolic conditions in the elderly and the need to maintain stable and effective disease control in this vulnerable population (Ebrahimpur et al. 2024, Zhou et al. 2025).

Despite the clear evidence of frequent risks related to AMIs, there remains a paucity of longitudinal studies assessing their long-term clinical impact. The few available studies suggest the high number of chronic conditions and medications serving as key risk factors (Onder et al. 2002, Holton et al. 2019a). In any case, the limited, yet existent body of research highlights a significant issue concerning health literacy, representing a significant barrier to prevention and management (Okan et al. 2020). On the one hand, it appears evident that healthcare professionals may not adequately address the interplay between alcohol consumption and medication use, confirming the need to increase medical education on the negative consequences related to alcohol and other substances use (Muzyk et al. 2020). On the other hand, patients—particularly older adults—and their caregivers need more thorough education about the potential risks associated with the concurrent use of alcohol and medications. This is even more significant because the rate of severe effects deriving from AMIs appears to be relevant and increasing over time (Zanjani et al. 2016, Holton et al. 2019a). Considering these findings, healthcare providers should prioritize routine screening for alcohol use among older adults, particularly those on multiple medications (Han and Moore 2018).

This review incorporates the latest literature, allowing to capture new insights and recent studies that may have been overlooked in prior works. It places particular emphasis on age-related physiological changes that may impact pharmacokinetics and pharmacodynamics, and how these changes impact alcohol and medications’ use. Finally, it offers insights for enhancing clinical practice and guiding future research. This study synthesizes data specifically on older adults, highlighting their vulnerabilities and offering a clear evidence-based perspective on the impact of AMIs in this population. By consolidating evidence and emphasizing age-specific risks, it provides a foundation for both clinical interventions and policy development. The strength of this review is constituted by the attempt to incorporate evidence-based guidelines into clinical practice and develop standardized lists of medications with potential AMIs, thereby helping to mitigate such risks (Knox et al. 2019, MacKillop et al. 2022). We believe this review lays out the groundwork for a future systematic review and highlights key areas where further clinical studies are needed. Future research should focus on longitudinal studies assessing the real-world consequences of AMIs to inform targeted interventions and policy recommendations.

## Conclusions

The findings of this review confirm that AMIs are common and can lead to severe consequences for older adults. Accordingly, professionals involved in the treatment of older people should always investigate alcohol use, especially when prescribing medications that may interact with alcohol, to reduce the risk of falls and other potential negative consequences.

## Supplementary Material

Supplementary_Table_1_FinalVersion_agag018

## Data Availability

The data associated with the current study are available from the corresponding author upon reasonable request.

## References

[ref1] Adams WL . Interactions between alcohol and other drugs. Int J Addict. 1995a;30:1903–23. 10.3109/10826089509071060 8751323

[ref2] Adams WL . Potential for adverse drug-alcohol interactions among retirement community residents. J Am Geriatr Soc. 1995b;43:1021–5. 10.1111/j.1532-5415.1995.tb05567.x 7657918

[ref3] Agabio R, Pisanu C, Gessa GL. et al. Sex differences in alcohol use disorder. Curr Med Chem. 2017;24:2661–70. 10.2174/0929867323666161202092908 27915987

[ref4] Aira M, Hartikainen S, Sulkava R. Community prevalence of alcohol use and concomitant use of medication—a source of possible risk in the elderly aged 75 and older? Int J Geriatr Psychiatry. 2005;20:680–5. 10.1002/gps.1340 16021662

[ref5] American Psychiatric Association . *Diagnostic and Statistical Manual of Mental Disorders, 5th Edition, Text Rev*. American Psychiatric Association Publishing, Washington, DC, 2022. 10.1176/appi.books.9780890425787.

[ref6] Anderson P, Scafato E, Galluzzo L. et al. Alcohol and older people from a public health perspective. Ann Ist Super Sanita. 2012;48:232–47. 10.4415/ANN_12_03_04 23007048

[ref7] Anton PE, Maphis NM, Linsenbardt DN. et al. Excessive alcohol use as a risk factor for Alzheimer's disease: epidemiological and preclinical evidence. Adv Exp Med Biol. 2025;1473:211–42. 10.1007/978-3-031-81908-7_10 40128481 PMC12720481

[ref8] Bebia Z, Buch SC, Wilson JW. et al. Bioequivalence revisited: influence of age and sex on CYP enzymes. Clin Pharmacol Ther. 2004;76:618–27. 10.1016/j.clpt.2004.08.021 15592333

[ref9] Breslow RA, Dong C, White A. Prevalence of alcohol-interactive prescription medication use among current drinkers: United States, 1999 to 2010. Alcohol Clin Exp Res. 2015;39:371–9. 10.1111/acer.12633 25597432 PMC4331237

[ref10] Cousins G, Galvin R, Flood M. et al. Potential for alcohol and drug interactions in older adults: evidence from the Irish longitudinal study on ageing. BMC Geriatr. 2014;14:57. 10.1186/1471-2318-14-57 24766969 PMC4008399

[ref11] Cruz-Jentoft AJ, Volkert D. Malnutrition in older adults. N Engl J Med. 2025;392:2244–55. 10.1056/NEJMra2412275 40499173

[ref12] Danpanichkul P, Duangsonk K, Tham EKJ. et al. Increased mortality from alcohol use disorder, alcohol-associated liver disease, and liver cancer from alcohol among older adults in the United States: 2000 to 2021. Alcohol Clin Exp Res (Hoboken). 2025;49:368–78. 10.1111/acer.15516 39701596 PMC11828968

[ref13] Del Río MC, Prada C, Alvarez FJ. The use of medication and alcohol among the Spanish population. Br J Clin Pharmacol. 1996;41:253–5. 10.1111/j.1365-2125.1996.tb00193.x 8866929

[ref14] Del Río MC, Prada C, Alvarez FJ. Do Spanish patients drink alcohol while undergoing treatment with benzodiazepines? Alcohol. 2002;26:31–4. 10.1016/s0741-8329(01)00195-1 11958944

[ref15] Delara M, Murray L, Jafari B. et al. Prevalence and factors associated with polypharmacy: a systematic review and meta-analysis. BMC Geriatr. 2022;22:601. 10.1186/s12877-022-03279-x Erratum in: BMC Geriatr. 2022 Sep 12;22(1):742. doi:10.1186/s12877-022-03388-7 35854209 PMC9297624

[ref16] Dharia SP, Slattum PW. Alcohol, medications, and the older adult. Consult Pharm. 2011;26:837–44. 10.4140/TCP.n.2011.837 22079793

[ref17] Diaz T, Strong KL, Cao B. et al. A call for standardised age-disaggregated health data. Lancet Healthy Longev. 2021; 2:e436–43. 10.1016/S2666-7568(21)00115-X Erratum in: Lancet Healthy Longev. 2021 Aug;2(8):e458. http://dx.doi.org/10.1016/S2666-7568(21)00171-9 34240065 PMC8245325

[ref18] Du Y, Scheidt-Nave C, Knopf H. Use of psychotropic drugs and alcohol among non-institutionalised elderly adults in Germany. Pharmacopsychiatry. 2008;41:242–51. 10.1055/s-0028-1083791 19067262

[ref19] Ebrahimpur M, Mohammadi-Vajari E, Sharifi Y. et al. Evaluation of the prevalence of cardiometabolic disorders (diabetes, hypertension, and hyperlipidemia) diagnosed, undiagnosed, treated, and treatment goal in the elderly: Bushehr elderly health program (BEH). BMC Endocr Disord. 2024;24:29. 10.1186/s12902-024-01561-0 38443972 PMC10913629

[ref20] Forster LE, Pollow R, Stoller EP. Alcohol use and potential risk for alcohol-related adverse drug reactions among community-based elderly. J Community Health. 1993;18:225–39. 10.1007/BF01324433 8408752

[ref21] Fraser AG . Pharmacokinetic interactions between alcohol and other drugs. Clin Pharmacokinet. 1997;33:79–90. 10.2165/00003088-199733020-00001 9260032

[ref22] García-Suástegui WA, Ramos-Chávez LA, Rubio-Osornio M. et al. The role of CYP2E1 in the drug metabolism or bioactivation in the brain. Oxid Med Cell Longev. 2017;2017:4680732. 10.1155/2017/4680732 28163821 PMC5259652

[ref23] GBD 2016 Alcohol Collaborators . Alcohol use and burden for 195 countries and territories, 1990-2016: a systematic analysis for the global burden of disease study 2016. Lancet. 2018; 392:1015–35. 10.1016/S0140-6736(18)31310-2 Erratum in: Lancet. 2018 Sep 29;392(10153):1116. http://dx.doi.org/10.1016/S0140-6736(18)32338-9. Erratum in: Lancet. 2019 Jun 22;393(10190):e44. http://dx.doi.org/10.1016/S0140-6736(19)31050-5 30146330 PMC6148333

[ref25] Gorsen SL, Mehuys E, De Bolle L. et al. Prevalence of alcohol-drug interactions in community-dwelling older patients with polypharmacy. Drug Metab Pers Ther. 2021;36:281–8. 10.1515/dmpt-2020-0183 34821122

[ref26] Grant BF, Goldstein RB, Saha TD. et al. Epidemiology of DSM-5 alcohol use disorder: results from the National Epidemiologic Survey on alcohol and related conditions III. JAMA Psychiatry. 2015;72:757–66. 10.1001/jamapsychiatry.2015.0584 26039070 PMC5240584

[ref27] Haight M, Smith P, Bray N. et al. Alcohol consumption among older adults in the United States amidst the COVID-19 pandemic: an analysis of the 2017-2021 Behavioral risk factor surveillance system. J Osteopath Med. 2024;125:95–100. 10.1515/jom-2024-0054 39072478

[ref28] Han BH, Moore AA. Prevention and screening of unhealthy substance use by older adults. Clin Geriatr Med. 2018;34:117–29. 10.1016/j.cger.2017.08.005 29129212 PMC5718360

[ref29] Heuberger RA . Alcohol and the older adult: a comprehensive review. J Nutr Elder. 2009;28:203–35. 10.1080/01639360903140106 21184367

[ref30] Holton AE, Gallagher P, Fahey T. et al. Concurrent use of alcohol interactive medications and alcohol in older adults: a systematic review of prevalence and associated adverse outcomes. BMC Geriatr. 2017a;17:148. 10.1186/s12877-017-0532-2 28716004 PMC5512950

[ref31] Holton AE, Gallagher PJ, Ryan C. et al. Consensus validation of the POSAMINO (POtentially serious alcohol-medication INteractions in older adults) criteria. BMJ Open. 2017b;7:e017453. 10.1136/bmjopen-2017-017453 PMC5695415 29122794

[ref32] Holton A, Boland F, Gallagher P. et al. Longitudinal prevalence of potentially serious alcohol-medication interactions in community-dwelling older adults: a prospective cohort study. Eur J Clin Pharmacol. 2019a;75:569–75. 10.1007/s00228-018-02608-7 30569283

[ref33] Holton A, Boland F, Gallagher P. et al. Potentially serious alcohol-medication interactions and falls in community-dwelling older adults: a prospective cohort study. Age Age. 2019b;48:824–31. 10.1093/ageing/afz112 PMC6814088 31579905

[ref34] Ilomäki J, Korhonen MJ, Enlund H. et al. Risk drinking behavior among psychotropic drug users in an aging Finnish population: the FinDrink study. Alcohol. 2008;42:261–7. 10.1016/j.alcohol.2008.02.002 18400450

[ref35] Ilomäki J, Gnjidic D, Hilmer SN. et al. Psychotropic drug use and alcohol drinking in community-dwelling older Australian men: the CHAMP study. Drug Alcohol Rev. 2013;32:218–22. 10.1111/j.1465-3362.2012.00496.x 22882728

[ref36] Immonen S, Valvanne J, Pitkälä KH. The prevalence of potential alcohol-drug interactions in older adults. Scand J Prim Health Care. 2013;31:73–8. 10.3109/02813432.2013.788272 23621352 PMC3656398

[ref37] Johnson BA, Seneviratne C. Alcohol-medical drug interactions. Handb Clin Neurol. 2014;125:543–59. 10.1016/B978-0-444-62619-6.00031-8 25307595

[ref38] Kirchheiner J, Seeringer A, Godoy AL. et al. CYP2D6 in the brain: genotype effects on resting brain perfusion. Mol Psychiatry. 2011;16:333–41. 10.1038/mp.2010.42 20368706

[ref39] Knox J, Hasin DS, Larson FRR. et al. Prevention, screening, and treatment for heavy drinking and alcohol use disorder. Lancet Psychiatry. 2019;6:1054–67. 10.1016/S2215-0366(19)30213-5 31630982 PMC6883141

[ref40] Lagnaoui R, Moore N, Dartigues JF. et al. Benzodiazepine use and wine consumption in the French elderly. Br J Clin Pharmacol. 2001;52:455–6. 10.1046/j.0306-5251.2001.01456.x 11678791 PMC2014578

[ref41] Lehner T, Gao B, Mackowiak B. Alcohol metabolism in alcohol use disorder: a potential therapeutic target. Alcohol Alcohol. 2024;59:agad077. 10.1093/alcalc/agad077. Erratum in: Alcohol Alcohol. 2024 Jul 21;59(5):agae064. http://dx.doi.org/10.1093/alcalc/agae064 37950904 PMC10783952

[ref42] Lu Y, Cederbaum AI. Cytochrome P450s and alcoholic liver disease. Curr Pharm Des. 2018;24:1502–17. 10.2174/1381612824666180410091511 29637855 PMC6053342

[ref43] MacKillop J, Agabio R, Feldstein Ewing SW. et al. Hazardous drinking and alcohol use disorders. Nat Rev Dis Primers. 2022;8:80. 10.1038/s41572-022-00406-1 Erratum in: Nat Rev Dis Primers. 2024 Sep 20;10(1):69. doi:10.1038/s41572-024-00561-7 36550121 PMC10284465

[ref44] Mann A, Miksys SL, Gaedigk A. et al. The neuroprotective enzyme CYP2D6 increases in the brain with age and is lower in Parkinson's disease patients. Neurobiol Aging. 2012;33:2160–71. 10.1016/j.neurobiolaging.2011.08.014 21958961

[ref45] Mao EH, Bu YL, Liu QL. et al. Alcohol consumption may not be a risk factor for sarcopenia in the older adults. Exp Biol Med (Maywood). 2025;250:10520. 10.3389/ebm.2025.10520 40510244 PMC12160937

[ref46] Meier P, Seitz HK. Age, alcohol metabolism and liver disease. Curr Opin Clin Nutr Metab Care. 2008;11:21–6. 10.1097/MCO.0b013e3282f30564 18090653

[ref47] Miksys S, Rao Y, Hoffmann E. et al. Regional and cellular expression of CYP2D6 in human brain: higher levels in alcoholics. J Neurochem. 2002;82:1376–87. 10.1046/j.1471-4159.2002.01069.x 12354285

[ref48] Minozzi S, La Rosa GRM, Salis F. et al. Combined pharmacological and psychosocial interventions for alcohol use disorder. Cochrane Database Syst Rev. 2025;2025:CD015673. 10.1002/14651858.CD015673.pub2 PMC11924338 40110869

[ref49] Moore AA, Whiteman EJ, Ward KT. Risks of combined alcohol/medication use in older adults. Am J Geriatr Pharmacother. 2007; 5:64–74. 10.1016/j.amjopharm.2007.03.006 17608249 PMC4063202

[ref50] Muzyk A, Smothers ZPW, Andolsek KM. et al. Interprofessional substance use disorder education in health professions education programs: a scoping review. Acad Med. 2020;95:470–80. 10.1097/ACM.0000000000003053 31651435

[ref51] National Institute on Alcohol Abuse and Alcoholism (NIAAA) . Helping Patients who Drink too Much: a Clinician’s Guide. 2005. s21151.pcdn.co/wp-content/uploads/2016/11/Helping-Patients-who-Drink-Too-Much_A-Clinicians-Guide-2017.pdf.

[ref52] National Survey on Drug Use and Health . U.S. Department of Health and Human Services, Substance Abuse and Mental Health Services Administration, Center for Behavioral Health Statistics and Quality. National Survey on Drug Use and Health, 2020 2002e2019. Rockville, MD, USA.

[ref53] Okan O, Rowlands G, Sykes S. et al. Shaping alcohol health literacy: a systematic concept analysis and review. Health Lit Res Pract. 2020;4:e3–e20. 10.3928/24748307-20191104-01. Erratum in: Health Lit Res Pract. 2020 Feb 11;4(1):e66. http://dx.doi.org/10.3928/24748307-20200116-01, http://dx.doi.org/10.3928/24748307-20200116-01 31935296 PMC6960007

[ref54] Onder G, Landi F, Della Vedova. et al. Moderate alcohol consumption and adverse drug reactions among older adults. Pharmacoepidemiol Drug Saf. 2002;11:385–92. 10.1002/pds.721 12271880

[ref55] Ortolá R, Sotos-Prieto M, García-Esquinas E. et al. Alcohol consumption patterns and mortality among older adults with health-related or socioeconomic risk factors. JAMA Netw Open. 2024;7:e2424495. 10.1001/jamanetworkopen.2024.24495 39133491 PMC11320169

[ref56] Pringle KE, Ahern FM, Heller DA. et al. Potential for alcohol and prescription drug interactions in older people. J Am Geriatr Soc. 2005;53:1930–6. 10.1111/j.1532-5415.2005.00474.x 16274374

[ref57] Qato DM, Manzoor BS, Lee TA. Drug-alcohol interactions in older U.S. Adults J Am Geriatr Soc. 2015;63:2324–31. 10.1111/jgs.13787 26503899 PMC4674266

[ref58] Ravindranath V, Boyd MR. Xenobiotic metabolism in brain. Drug Metab Rev. 1995;27:419–48. 10.3109/03602539508998330 8521749

[ref59] Rigler SK . Alcoholism in the elderly. Am Fam Physician. 2000;61:1710–1716. 10750878

[ref60] Schröder S, Westhoff MS, Pfister T. et al. Drug safety in older patients with alcohol use disorder: a retrospective cohort study. Ther Adv Psychopharmacol. 2024; 14:20451253241232563. 10.1177/20451253241232563 38384595 PMC10880528

[ref61] Sheahan SL, Coons SJ, Robbins CA. et al. Psychoactive medication, alcohol use, and falls among older adults. J Behav Med. 1995;18:127–40. 10.1007/BF01857865 7563042

[ref62] Silva SM, Madeira MD, Ruela C. et al. Prolonged alcohol intake leads to irreversible loss of vasopressin and oxytocin neurons in the paraventricular nucleus of the hypothalamus. Brain Res. 2002;925:76–88. 10.1016/s0006-8993(01)03261-9 11755902

[ref63] Silva-Adaya D, Garza-Lombó C, Gonsebatt ME. Xenobiotic transport and metabolism in the human brain. Neurotoxicology. 2021;86:125–38. 10.1016/j.neuro.2021.08.004 34371026

[ref64] Soltani S, Jayedi A, Ghoreishy S. et al. Alcohol consumption and frailty risk: a dose-response meta-analysis of cohort studies. Age Age. 2024;53:afae199. 10.1093/ageing/afae199 39300899

[ref65] Sudhinaraset M, Wigglesworth C, Takeuchi DT. Social and cultural contexts of alcohol use: influences in a social-ecological framework. Alcohol Res. 2016;38:35–45. 10.35946/arcr.v38.1.05 27159810 PMC4872611

[ref66] Tevik K, Bergh S, Selbæk G. et al. A systematic review of self-report measures used in epidemiological studies to assess alcohol consumption among older adults. PLoS One. 2021;16: e0261292. 10.1371/journal.pone.0261292 34914759 PMC8675766

[ref67] Traccis F, Presciuttini R, Pani PP. et al. Alcohol-medication interactions: a systematic review and meta-analysis of placebo-controlled trials. Neurosci Biobehav Rev. 2022;132:519–41. 10.1016/j.neubiorev.2021.11.019 34826511

[ref68] Tschorn M, Schulze S, Förstner BR. et al. Predictors and prevalence of hazardous alcohol use in middle-late to late adulthood in Europe. Aging Ment Health. 2023;27:1001–10. 10.1080/13607863.2022.2076208 35639449

[ref69] Veldhuizen S, Wade TJ, Cairney J. Alcohol consumption among Canadians taking benzodiazepines and related drugs. Pharmacoepidemiol Drug Saf. 2009;18:203–10. 10.1002/pds.1702 19115421

[ref70] Weathermon R, Crabb DW. Alcohol and medication interactions. Alcohol Res Health. 1999;23:40–54. 10890797 PMC6761694

[ref71] White AM, Orosz A, Powell PA. et al. Alcohol and aging—an area of increasing concern. Alcohol. 2023;107:19–27. 10.1016/j.alcohol.2022.07.005 35940508

[ref72] Wong H, Heuberger R, Logomarsino J. et al. Associations between alcohol use, polypharmacy and falls in older adults. Nurs Older People. 2016;28:30–6. 10.7748/nop.28.1.30.s22 26938609

[ref73] Xu Q, Ou X, Li J. The risk of falls among the aging population: a systematic review and meta-analysis. Front Public Health. 2022;10:902599. 10.3389/fpubh.2022.902599 36324472 PMC9618649

[ref74] Zahr NM . Alcohol use disorder and dementia: a review. Alcohol Res. 2024;44:03. 10.35946/arcr.v44.1.03 38812709 PMC11135165

[ref75] Zanjani F, Smith R, Slavova S. et al. Concurrent alcohol and medication poisoning hospital admissions among older rural and urban residents. Am J Drug Alcohol Abuse. 2016;42:422–30. 10.3109/00952990.2016.1154966 27184414 PMC4998842

[ref76] Zarezadeh M, Mahmoudinezhad M, Faghfouri AH. et al. Alcohol consumption in relation to cognitive dysfunction and dementia: a systematic review and dose-response meta-analysis of comparative longitudinal studies. Ageing Res Rev. 2024;100:102419. 10.1016/j.arr.2024.102419 39038743

[ref77] Zhou K, Wang A, Yi K. Cardiometabolic multimorbidity and frailty in middle-aged and older adults: a cross-nationally harmonized study. Front Public Health. 2025;13:1565682. 10.3389/fpubh.2025.1565682 40308911 PMC12041071

