# Supplementary material for: Alcohol-medication interactions among older people: a narrative review on age-specific evidence
Source: Alcohol Alcohol. 2026 Apr 5;61(3):agag018. doi: 10.1093/alcalc/agag018 (PMC13050505; doi:10.1093/alcalc/agag018)
Supplement: Supplementary_Table_1_FinalVersion_agag018 [file supplementary_table_1_finalversion_agag018.docx]

**Supplementary Table 1 – Main Findings of the included studies**

| Study | Main findings |
| --- | --- |
| Adams,  1995a | High alcohol (≥70%) and ≥1 prescription drug use (60–78%) among adults ≥60 y; induction of CYP enzymes by chronic alcohol; increased risks of sedation, cognitive impairment, gastrointestinal bleeding (notably with NSAIDs), and hepatotoxicity. |
| Adams,  1995b | Concomitant alcohol and high-risk medication use (6% ≥7 drinks/week); frequent exposure to antihypertensives, aspirin, and NSAIDs; need for enhanced screening and public education. |
| Aira et al.,  2005 | Alcohol consumption in 44% of home-dwelling adults ≥75 y; persistence of clinically relevant theoretical interaction risks despite modest intake. |
| Anderson et al., 2012 | Responsiveness of older adults to alcohol interventions comparable to younger populations; paucity of age-specific PK and interaction data; need for longitudinal geriatric research. |
| Bebia et al.,  2004 | Age-related increase in CYP2E1 and decline in CYP2C19 activity; implications for altered drug metabolism and toxicity in adults ≥50 y. |
| Breslow et al.,  2015 | High prevalence of concurrent alcohol use with cardiovascular, CNS, and metabolic agents in U.S. population; substantial exposure to potential interactions. |
| Cousins et al.,  2014 | Sociodemographic predictors (male sex, younger age, urban residence, higher education, never-married status, and smoking) of moderate and heavy drinking and AI drug use in older Irish adults. |
| Del Río et al.,  1996 | High prevalence of daily alcohol consumption among antidepressant and respiratory drug users; support for mandatory drug–alcohol warning statements. |
| Del Río et al.,  2002 | Age-related increase in daily alcohol use among BZD users (peak 56–65 y); male predominance. |
| Dharia and Slattum, 2011 | Recommendation for routine alcohol screening (CAGE, MAST-G) in older adults, particularly those receiving warfarin or CNS depressants. |
| Du et al.,  2008 | High prevalence of psychotropic use and risky drinking in adults 60–79 y; association with polypharmacy, poor health, and social vulnerability. |
| Forster et al.,  1993 | Frequent concomitant alcohol use with OTC analgesics and antihypertensives; recommendation for universal screening of older drinkers. |
| Fraser,  1997 | Regular alcohol–medication co-use (25–38%); additive CNS depression with sedatives and synergistic GI toxicity with NSAIDs/aspirin; increased fall and bleeding risk. |
| García-Suástegui et al., 2017 | CYP2E1 induction by alcohol; oxidative injury in aging brain regions; relevance for neurodegenerative vulnerability. |
| Gorsen et al.,  2021 | High prevalence of contraindicated medication use among heavy/frequent drinkers ≥70 y; absence of safe alcohol thresholds with concurrent pharmacotherapy. |
| Holton et al.,  2017a | Prevalence of AI drug exposure (21–35%); association between moderate alcohol intake and increased ADR risk at admission. |
| Holton et al.,  2017b | Development of POSAMINO criteria for potentially serious AMIs in ≥65 y. |
| Holton et al.,  2019a | 18% prevalence of ≥1 serious alcohol–medication interaction in ≥65 y.; Younger age, male sex, polypharmacy, and multimorbidity predicted higher odds and counts of POSAMINO criteria. |
| Holton et al.,  2019b | CNS-related AMIs associated with increased overall and injurious fall risk over 4 years in adults ≥65 y. |
| Ilomäki et al.,  2008 | High prevalence of binge/heavy drinking among older Finnish men; increased risk of CNS depression and injury. |
| Ilomäki et al.,  2013 | Independent association between sedative/anxiolytic use and daily drinking; concern for additive sedation and dependence. |
| Immonen et al., 2013 | Sociodemographic predictors (male, younger, married, and higher educated) of at-risk alcohol use; association between alcohol–AI drug exposure and falls/injuries. |
| Kirchheiner et al., 2011 | Inverse association between CYP2D6 activity and thalamic perfusion; implications for CNS drug metabolism. |
| Lagnaoui et al., 2001 | Inverse association between heavy wine consumption and BDZ use in adults ≥65 y. |
| Lu et al.,  2018 | CYP2E1-mediated oxidative stress and hepatotoxicity in chronic alcohol exposure; increased age-related susceptibility. |
| Mann et al.,  2012 | Triphasic age-related CYP2D6 expression pattern; implications for altered CNS drug response in later life. |
| Meier and Seitz, 2008 | Age-related hepatic structural decline; ethanol–CYP2E1 interactions increasing hepatotoxic risk. |
| Miksys et al.,  2002 | Brain CYP2D6 expression across adulthood; potential alcohol-induced enhancement of local drug metabolism and neurotoxicity. |
| Moore et al.,  2007 | High prevalence of at-risk alcohol–medication combinations; age-related PK changes increasing BAL and drug half-life; association with falls and chronic disease. |
| Onder et al.,  2002 | Independent association between moderate alcohol intake and ADRs (headache, metabolic/endocrine ADRs) in older adults, particularly women. |
| Pringle et al.,  2005 | Common alcohol–AI combinations: NSAIDs, antihistamines, antihypertensives; sociodemographic predictors of co-use: younger age, male sex, and higher education predict concurrent use. |
| Qato et al.,  2015 | 20% prevalence of AMIs risk among older drinkers; Higher odds among men, non-Hispanic Whites, higher income/education, and multimorbid individuals. |
| Ravindranath et al., 1995 | Ethanol-inducible CYP expression in human brain; heterogeneous distribution and high antidepressant affinity. |
| Rigler,  2000 | High alcohol use in adults 60–94 y; age-related increase in peak ethanol levels and reduced hepatic clearance; standard screening tools (e.g., CAGE) insensitive for binge drinking in ≥60 y. |
| Schröder et al., 2024 | High prevalence of serious AMIs (80.7%) among older adults with AUD; frequent involvement of cardiovascular and CNS drugs. |
| Sheahan et al., 1995 | High prevalence of psychoactive drug use, alcohol consumption and falls among adults ≥ 55 y; independent association between psychoactive medication use and fall risk. |
| Silva et al.,  2002 | Chronic ethanol–induced neuronal loss and hypertrophy in aged rat PVN; persistence after withdrawal. |
| Silva-Adaya et al., 2021 | Age-related increase in MAO-B and decline in ALDH2; CYP2E1 hypomethylation and CYP2D6 reduction; shift toward oxidative neurotoxicity. |
| Traccis et al.,  2022 | Alcohol-induced increases in Cmax of several drugs; major evidence gaps in ≥65 y; recommendation to avoid alcohol during early pharmacotherapy. |
| Veldhuizen et al., 2009 | Reduced heavy drinking with increasing age among BZD users; low prevalence of hazardous drinking ≥60 y. |
| Weathermon et al., 1999 | Age-related PK changes (reduced gastric ADH, increased BAL); additive CNS depression and GI toxicity; heightened PK/PD vulnerability. |
| Wong et al.,  2016 | High polypharmacy prevalence in adults aged 60–103 y; medication use and polypharmacy independently associated with falls (not alcohol). |
| Zanjani et al.,  2016 | Marked increase in alcohol–medication hospitalizations ≥50 y; BZD frequently implicated; predominance of medication poisoning diagnoses. |

**Abbreviations**

ADR, Adverse Drug Reaction; ADH, Alcohol Dehydrogenase; ALDH, **Aldehyde Dehydrogenase 2;** AMIs, Alcohol–Medication Interactions; AI, Alcohol-Interacting; AUD, Alcohol Use Disorder; BAL, Blood Alcohol Level; BZD, Benzodiazepine; CAGE, Cut-down, Annoyed, Guilty, Eye-opener questionnaire; Cmax, Maximum Plasma Concentration
CNS, Central Nervous System; CYP, Cytochrome P450; GI, Gastrointestinal; MAO-B, Monoamine Oxidase B; MAST-G, Michigan Alcoholism Screening Test–Geriatric Version; NSAIDs, Nonsteroidal Anti-Inflammatory Drugs; OTC, Over-the-counter; PK, Pharmacokinetics; PD, Pharmacodynamics; POSAMINO, Potentially Serious Alcohol–Medication Interactions in Older Adults; PVN, Paraventricular Nucleus; Y, years
